# Caloric restriction lowers endocannabinoid tonus and improves cardiac function in type 2 diabetes

**DOI:** 10.1038/s41387-017-0016-7

**Published:** 2018-01-17

**Authors:** Huub J. van Eyk, Linda D. van Schinkel, Vasudev Kantae, Charlotte E. A. Dronkers, Jos J. M. Westenberg, Albert de Roos, Hildo J. Lamb, J. Wouter Jukema, Amy C. Harms, Thomas Hankemeier, Mario van der Stelt, Ingrid M. Jazet, Patrick C. N. Rensen, Johannes W. A. Smit

**Affiliations:** 1 Department of Medicine, Division of Endocrinology, Leiden University Medical Center (LUMC), Leiden, The Netherlands; 2 Einthoven Laboratory for Experimental Vascular Medicine, LUMC, Leiden, The Netherlands; 3 Division of Systems Biomedicine and Pharmacology, Leiden Academic Centre for Drug Research (LACDR), Leiden University, Leiden, The Netherlands; 4 Department of Radiology, LUMC, Leiden, The Netherlands; 5 Department of Cardiology, LUMC, Leiden, The Netherlands; 6 Department Molecular Physiology, Leiden Institute of Chemistry (LIC), Leiden University, Leiden, The Netherlands; 7 Department of Medicine, Radboud University Medical Center, Nijmegen, The Netherlands

## Abstract

**Background/Objectives:**

Endocannabinoids (ECs) are associated with obesity and ectopic fat accumulation, both of which play a role in the development of cardiovascular disease (CVD) in type 2 diabetes (T2D). The effect of prolonged caloric restriction on ECs in relation to fat distribution and cardiac function is still unknown. Therefore, our aim was to investigate this relationship in obese T2D patients with coronary artery disease (CAD).

**Subjects/Methods:**

In a prospective intervention study, obese T2D patients with CAD (*n* = 27) followed a 16 week very low calorie diet (VLCD; 450–1000 kcal/day). Cardiac function and fat accumulation were assessed with MRI and spectroscopy. Plasma levels of lipid species, including ECs, were measured using liquid chromatography-mass spectrometry.

**Results:**

VLCD decreased plasma levels of virtually all measured lipid species of the class of *N*-acylethanolamines including the EC anandamide (AEA; −15%, *p* = 0.016), without decreasing monoacylglycerols including the EC 2-arachidonoylglycerol (2-AG). Baseline plasma AEA levels strongly correlated with the volume of subcutaneous white adipose tissue (SAT; *R*^2^ = 0.44, *p* < 0.001). VLCD decreased the volume of SAT (−53%, *p* < 0.001), visceral white adipose tissue (VAT) (−52%, *p* < 0.001), epicardial white adipose tissue (−15%, *p* < 0.001) and paracardial white adipose tissue (−28%, *p* < 0.001). VLCD also decreased hepatic (−86%, *p* < 0.001) and myocardial (−33%, *p* < 0.001) fat content. These effects were accompanied by an increased left ventricular ejection fraction (54.8 ± 8.7–56.2 ± 7.9%, *p* = 0.016).

**Conclusions:**

Caloric restriction in T2D patients with CAD decreases AEA levels, but not 2-AG levels, which is paralleled by decreased lipid accumulation in adipose tissue, liver and heart, and improved cardiovascular function. Interestingly, baseline AEA levels strongly correlated with SAT volume. We anticipate that dietary interventions are worthwhile strategies in advanced T2D, and that reduction in AEA may contribute to the improved cardiometabolic phenotype induced by weight loss.

## Introduction

The endocannabinoid (EC) system is a key player in lipid and glucose metabolism and in regulation of energy balance^1^. It consists of the cannabinoid receptors type 1 (CB1R) and type 2 (CB2R), their ligands, the endocannabinoids (ECs), and the enzymes responsible for synthesis and degradation of ECs. The two most well-studied ECs, anandamide (AEA) and 2-arachidonoylglycerol (2-AG), belong to the class of *N*-acylethanolamines and monoacylglycerols, respectively. The ECs are phospholipid-derived lipids that can be produced by any cell type and organ and act mainly as paracrine mediators. Binding of ECs to the cannabinoid receptors results in various metabolic effects, including increased food intake, enhanced lipogenesis and reduced energy expenditure^2,3^. This is supported by several studies reporting strong associations between high plasma EC levels and triglycerides (TG), intra-abdominal obesity and insulin resistance in obese and type 2 diabetes (T2D) patients^4–7^. Furthermore, overactivity of the EC system can promote beta cell failure through activation of the Nlrp3-ASC inflammasome in infiltrating macrophages^8^. Thus, elevated levels of ECs can contribute to relative insulin deficiency and to accumulation of visceral adipose tissue (VAT) and obesity, and as such may have a causal role in the pathophysiology of T2D and CVD^3^.

The prevalence of T2D, which is associated with the Western obesogenic lifestyle, is increasing^9^. T2D patients have a two-fold increased risk to develop cardiovascular disease (CVD), their major cause of death^10^. Moreover, the 7-year incidence rate of myocardial infarction (MI) following a previous MI is increased more than two-fold in T2D patients compared to non-diabetic subjects^11^. Many risk factors play a role in the development of cardiovascular complications, and a major role is attributed to ectopic lipid deposition. Ectopic lipid deposition stimulates inflammation and causes insulin resistance, probably as a consequence of accumulation of toxic metabolites including diacylglycerol and ceramides that interfere with the insulin signaling process^12^. For example, lipid accumulation can occur in myocardial tissue and in the pericardial fat depot, which is associated with impaired myocardial function and increased risk and degree of coronary atherosclerosis^13–16^.

Many studies have targeted the EC system to improve metabolic health. Blockade of the CB1R with rimonabant caused weight loss and improved lipid metabolism in obese animal models and humans, but had unacceptable psychiatric side effects^17–20^. Alternatively, EC levels can be reduced by dietary interventions. For example, krill oil, which has a high polyunsaturated fatty acid (PUFA) content, reduces plasma 2-AG levels in obese humans, probably by reducing the availability of EC biosynthetic precursors^21^. Also, dietary long-chain PUFA reduces ectopic fat deposition in liver and heart and susceptibility for inflammation in Zucker rats^22, 23^, supporting a connection between the EC system and ectopic lipid deposition. Studies in humans assessing the effect of weight loss on plasma EC levels show variable results: while some studies showed a decrease in EC levels^6, 24^, other studies showed no effect^25, 26^. The effect of more pronounced weight loss by intensive caloric restriction on plasma EC levels is as yet unknown.

Previously our group showed that prolonged caloric restriction decreases ectopic fat deposition and improves myocardial function in obese T2D patients without CAD^27^. However, the effects of this intervention in T2D patients *with* established CAD are still unknown. Therefore, our aim was to investigate the effect of caloric restriction on plasma EC levels, ectopic lipid accumulation and cardiovascular function in obese patients with T2D and established CAD.

## Methods

### Subjects

Obese (BMI > 25 kg/m²) T2D patients with documented coronary artery disease (CAD) were recruited via advertisements and from the outpatient clinic of the Leiden University Medical Center (LUMC), Leiden, the Netherlands. Established atherosclerosis or CAD were defined as a history of MI and/or percutaneous coronary intervention and/or a > 50% stenosis in a coronary artery as documented by computed tomography angiography. Exclusion criteria were hepatic disease, glomerular filtration rate <60 mL/min, congenital heart disease and general contraindications to magnetic resonance (MR) scanning. Subjects underwent a medical screening including their medical history, physical examination, and blood chemistry tests. The study was approved by the local ethics committee and performed in accordance with the principles of the revised Declaration of Helsinki. Written informed consent was obtained from all subjects before participation. The study was registered in the Dutch Trial Register (NTR-2897).

### Study design

The study was conducted at the LUMC, Leiden, the Netherlands. Patients were studied on two occasions: at baseline and after a 16-week dietary intervention period, during which a VLCD was prescribed. In order to assess the variability in study parameters without dietary intervention, 13 of the 27 patients were also studied 16 weeks prior to the start of the VLCD. Patients were instructed not to alter lifestyle habits during the study.

The VLCD consisted of Prodimed products (Prodimed® Benelux BV, Valkenswaard, the Netherlands), which are low in calories and have a relatively high protein content of 67% and a low fat content of 5%. All patients started with total meal replacement: 4–6 sachets a day (400–600 kcal/day) including a warm meal of Prodimed for three weeks, supplemented with a limited choice of vegetables. After these three weeks caloric intake was increased, by replacing a Prodimed at dinner time by meat or fish. Afterwards, when an additional 3% weight loss was achieved, the caloric intake was further expanded with one Prodimed being replaced with a normal meal. One week before the last study day, the diet was expanded, with a normal breakfast to achieve a caloric intake of 1000 kcal/day. Use of sulphonyl urea derivatives was discontinued the day the VLCD started and insulin therapy was adjusted according to glucose levels. Patients on insulin treatment were asked to measure their blood glucose levels 4 times a day throughout the study.

During the intervention period, patients visited the outpatient clinic weekly, and blood pressure and body weight were measured. Blood was drawn monthly to assess safety parameters. At each study day, anthropometric measurements, blood sampling and MR imaging and spectroscopy were performed after ≥5 h of fasting. Blood was centrifuged at 4 °C and EDTA plasma and serum were stored at −80 °C until analyzses.

### Biochemical assays

Serum concentrations of glucose, total cholesterol, HDL-cholesterol and TG were measured on a Modular P800 analyzer (Roche Diagnostics, Mannheim, Germany), NEFA were measured using a commercial kit (Wako Chemicals, Neuss, Germany), and insulin on an Immulite 2500 analyzer (Siemens, Breda, the Netherlands). LDL-cholesterol was calculated according to Friedewald’s formula^28^. HbA_1c_ was measured on an HPLC system (Roche Diagnostics, Mannheim, Germany). High-sensitivity CRP (hs-CRP) levels were assessed using precoated 96-well multisport plates from Meso Scale Discovery (Gaithersburg, Maryland, USA). Concentrations of ALT and AST were measured with the Cobas Integra 800 analyzer (Roche Diagnostics, Mannheim, Germany). Plasma levels of several lipid species including ECs were quantified using liquid chromatography coupled with tandem mass spectrometry (LC-MS/MS) as described previously^29^.

### MR spectroscopy and imaging

MR measurements were performed at a 1.5-Tesla MR-scanner (Gyroscan ACS-NT15; Philips Medical Systems, Best, the Netherlands).

#### Adipose tissue depots

The volume of pericardial fat, consisting of both epicardial and paracardial fat, was derived from fat-selective imaging using spectral presaturation with inversion recovery, as described before^30^. Abdominal VAT and subcutaneous adipose tissue (SAT) volumes were quantified at the level of the fifth lumbar vertebra, using a turbo spin echo imaging sequence, as described before^15^. Volumes of VAT and SAT were quantified using MASS.

#### MR spectroscopy

Proton MR spectroscopy (^1^H-MRS) was performed to quantify myocardial and hepatic TG content. Details on acquisition and post processing were described previously^31, 32^. For the liver, voxel sites were matched at all study occasions.

#### Delayed enhancement

Delayed enhancement MRI for detection of myocardial scar was performed 15 min after injection of gadetorate meglumine (0.3 mL/kg, Dotarem; Guerbet, Bloomington, USA) as described^33^.

#### Left ventricular dimensions and function

The heart was imaged in short-axis orientation to assess systolic function, as previously described^34^. Left ventricular (LV) end-diastolic and end-systolic contours were drawn, using in-house developed validated MASS software (LUMC, Leiden, the Netherlands). LV end-diastolic volume, end-systolic volume, ejection fraction, mass, cardiac output and stroke volume were calculated. Several function parameters were indexed to body surface area. LV mass was divided by LV end-diastolic volume to obtain the LV mass/LV end-diastolic volume ratio, also known as concentricity. LV diastolic function was studied from transmitral flow rate graphs, assessed from 3D three-directional velocity encoded MRI with retrospective valve tracking as previously described^35^. From the transmitral flow rate graphs, the following LV diastolic function parameters were determined using MASS software: maximal flow velocities and peak filling rate in early diastole and at diastolic atrial contraction. The ratio between peak filling at early diastole and diastolic atrial contraction was calculated. In addition, the downslope after early peak filling rate (deceleration) and the ratio between maximal flow velocity during early diastole and the through-plane velocity assessed in the myocardial wall, which is the estimate of the LV filling pressure, were assessed^36, 37^.

#### Pulse wave velocity

To evaluate aortic stiffness, aortic pulse wave velocity (PWV) was determined, using in-house developed and validated Matlab software (LUMC, Leiden, The Netherlands) as previously described^38^. A scout view of the aorta was obtained. Subsequently, two time-resolved velocity-encoded acquisitions perpendicular to the ascending aorta at the level of the pulmonary trunk and at the level of the aortic bifurcation were assessed, resulting in through-plane flow measurements. PWV was calculated with the formula: Δ*x*/Δ*t*. Δ*x* is the length of the aorta between two measurement sites and Δ*t* is the time delay between the arrivals of the foot of the pulse wave at the respective measurements site. The distance between the measurement sites was determined manually with MASS. MASS and FLOW (LUMC, Leiden, the Netherlands) were used for data analysis.

### Statistical analysis

Data are presented as mean ± SD or as median (interquartile range (IQR)) when not normally distributed. Based on the previous 16 week VLCD study we performed in obese patients with T2DM without cardiovascular complications, we performed a sample size calculation^27^. A *p* value < 0.05 was considered statistically significant. Changes within participants were assessed using paired sample t-tests or Wilcoxon signed-rank test when appropriate. Linear regression analysis computed by Pearson’s correlation was performed to identify correlations between plasma EC levels and various metabolic parameters. Statistical analyzes were performed with SPSS for Windows, version 23.0 (SPSS Inc., Chicago, USA).

## Results

### Clinical characteristics

While 32 patients were initially included to receive VLCD intervention, two patients left the study due to intolerance to the diet, one patient left because of worsening of Ménière’s disease, and two patients were excluded due to non-adherence to the diet. Of the 27 participants who completed the study, results are shown in Table 1. Individuals were on average 62.2 ± 6.0 years old, had a body weight of 98.5 ± 16.0 kg, a BMI of 32.2 ± 4.7 kg/m^2^, and most of them were male (82%). VLCD induced an average weight loss of 16.5 ± 5.5 kg (*p* < 0.001), and BMI decreased on average with 5.4 ± 1.7 kg/m^2^ (*p* < 0.001). VLCD improved glycemic control, as reflected by decreased fasting plasma glucose levels and decreased HbA_1c_ levels. VLCD decreased ALT, decreased total cholesterol and TG, and increased HDL-cholesterol, without changing NEFA. In addition, VLCD reduced systolic and diastolic blood pressures and heart rate.

**Table 1:** Clinical and metabolic characteristics of obese type 2 diabetes patients with documented coronary artery disease before and after very low calorie diet

	Before VLCD	After VLCD	*p* value
*Clinical characteristics*			
Age (years)	62.2 ± 6.0		
Males, *n* (%)	22 (82%)		
T2D duration (years)	11.0 ± 8.5		
Patients on insulin, n (%)	12 (44%)	9 (33%)	
Insulin dose (units/day)	79 ± 36	16 ± 12	
Body weight (kg)	98.5 ± 16.0	82.0 ± 14.0	<0.001
BMI (kg/m^2^)	32.2 ± 4.7	26.8 ± 4.1	<0.001
Waist circumference (cm)	111 ± 12	93 ± 11	<0.001
Systolic blood pressure (mmHg)	146 ± 14	129 ± 12	<0.001
Diastolic blood pressure (mmHg)	83 ± 10	75 ± 9	0.002
Heart rate (beats/min)	68 ± 12	60 ± 8	0.002
*Metabolic characteristics*			
Glucose (mmol/L)	7.4 ± 1.7	6.3 ± 1.2	0.002
HbA_1c_ (%)	6.9 ± 0.9	5.8 ± 0.5	<0.001
HbA_1c_ (mmol/mol)	51 ± 10	40 ± 6	<0.001
Triglycerides (mmol/L)	1.80 ± 0.89	1.17 ± 0.43	<0.001
Total cholesterol (mmol/L)	4.18 ± 0.86	3.82 ± 0.67	0.018
HDL-cholesterol (mmol/L)	1.18 ± 0.28	1.32 ± 0.34	0.001
LDL-cholesterol (mmol/L)	2.18 ± 0.71	1.97 ± 0.60	0.088
NEFA (mmol/L)	0.61 ± 0.25	0.59 ± 0.29	0.836
ALT (IU/L)	30 ± 10	24 ± 11	0.024
AST (IU/L)	25 ± 7	22 ± 7	0.057
hs-CRP (mg/L)	1.7 (4.5)	1.1 (3.0)	0.107

Data are means ± SD or median (interquartile range), *n* = 27. *p* value before vs. after diet based on a paired sample t-test or Wilcoxon signed-rank test

*ALT* alanine transaminase, *AST* aspartate transaminase, *hs-CRP* high-sensitivity C-reactive protein, *IU* International unit

Sixteen of these 27 patients had previously experienced a MI, 24 underwent percutaneous coronary intervention and three patients had a coronary artery bypass grafting. One patient had >50% occlusion of the coronary arteries on computed tomography angiography without a subsequent intervention. There were no differences in age, duration of T2D or anthropometric measurements between patients with and without a MI in medical history. At baseline 12 patients used insulin and all patients were on oral antihyperglycemic drugs. During the VLCD, insulin treatment was discontinued in 3 patients and in three patients all glucose lowering drugs could be discontinued. As shown in Supplementary Table 1, clinical and metabolic parameters of the subgroup of 13 patients that was studied 16 weeks prior to the start of the VLCD did not change during this period. Also, MR parameters showed no significant variability before start of VLCD, as shown in Supplementary Table 2.

### Endocannabinoid levels

**Fig. 1:**
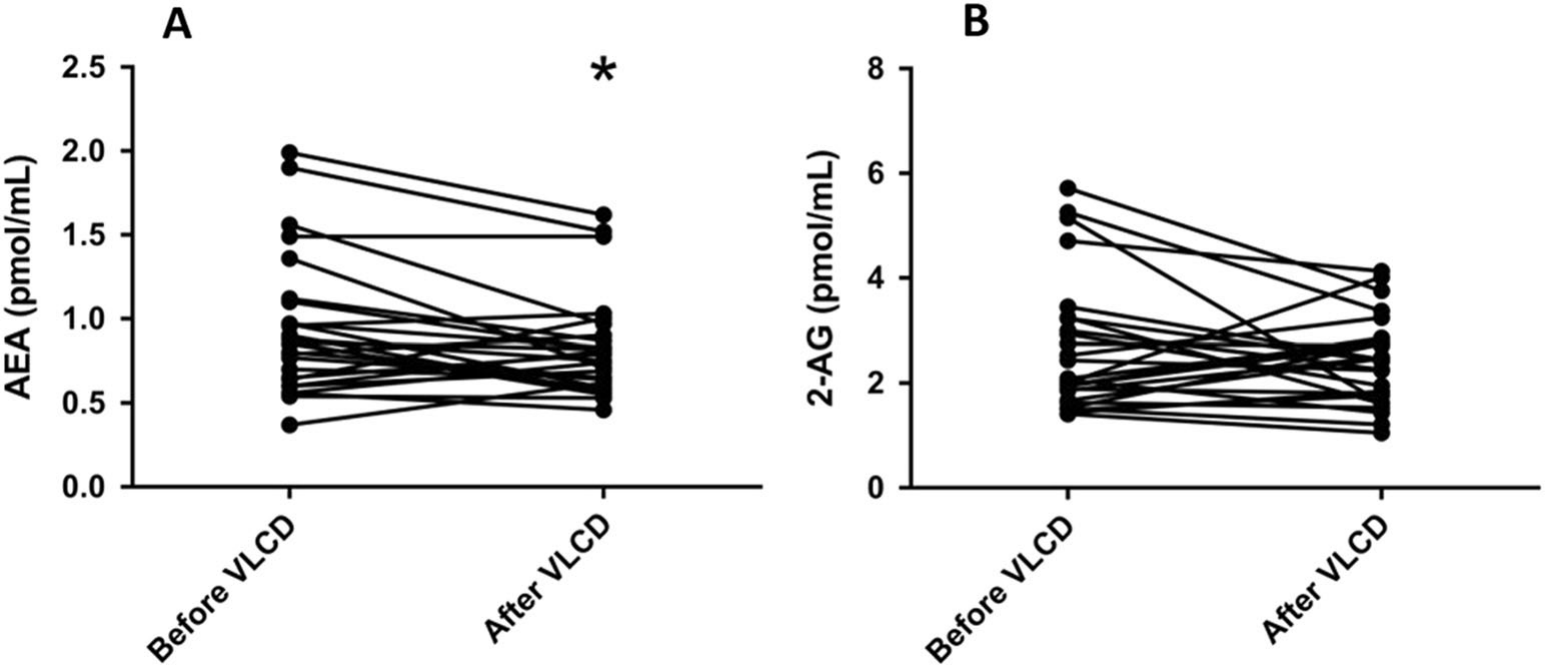
Plasma levels of AEA and 2-AG before and after VLCD Change in AEA (**a**) and 2-AG (**b**). *p < 0.05 vs. before VLCD, *n* = 27

**Fig. 2:**
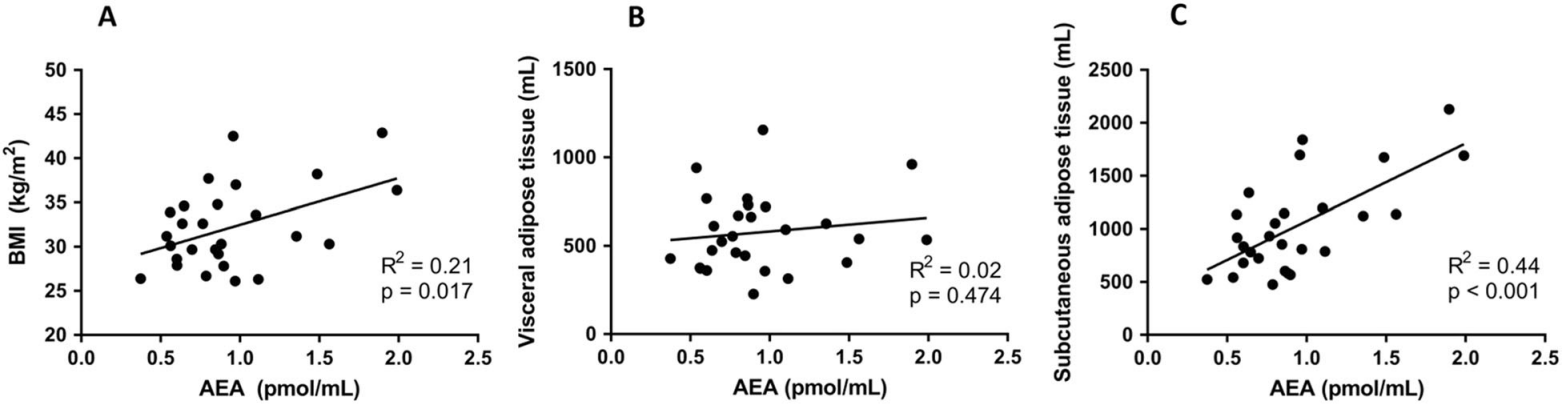
Correlations of AEA with BMI and adipose tissue volume AEA plasma levels in relation to BMI (**a**), VAT volume (**b**), and SAT volume (**c**) as measured at baseline, *n* = 27

The effects of the VLCD on plasma levels of the various ECs and lipid species are summarized in Table 2. VLCD decreased virtually all ECs from the group of *N*-acylethanolamines, without decreasing any ECs belonging to the group of monoacylglycerols. With respect to the most well studied CBR ligands, VLCD decreased plasma AEA levels (−13%; *p* = 0.024), without significantly decreasing plasma 2-AG levels (Table 2; Fig. 1). At baseline, plasma 2-AG levels did not correlate with either BMI, VAT and SAT volume (not shown). Interestingly, baseline plasma AEA levels correlated positively with BMI (*R*^2^ = 0.21, *p* = 0.017) and, while plasma AEA levels did not correlate with VAT volume, they strongly correlated positively with SAT volume (*R*^2^ = 0.44, *p* < 0.001) (Fig. 2**)**. Plasma levels of 2-AG, but not AEA, correlated with TG levels (*R*^2^ = 0.41, *p* < 0.001). Changes in plasma AEA and 2-AG levels as induced by the VLCD did not correlate with changes in adipose tissue volumes and parameters of cardiac function (Supplement Table 3).

**Table 2:** Plasma levels of lipid species before and after very low calorie diet

	Before VLCD	After VLCD	*p* value
*N-acylethanolamines (pmol/mL)*			
AEA	0.94 ± 0.41	0.82 ± 0.30	0.024
PEA	7.57 ± 2.03	6.26 ± 1.28	<0.001
SEA	6.36 ± 1.79	4.88 ± 0.87	<0.001
POEA	0.55 ± 0.47	0.34 ± 0.24	0.003
OEA	5.13 ± 1.57	4.74 ± 1.17	0.091
LEA	2.52 ± 0.64	2.13 ± 0.38	0.001
Alpha-LEA	0.19 ± 0.06	0.15 ± 0.03	0.003
DGLEA	0.16 ± 0.08	0.12 ± 0.04	<0.001
PDEA	0.09 ± 0.03	0.08 ± 0.02	0.105
EPEA	0.60 ± 0.37	0.37 ± 0.24	0.001
DEA	0.28 ± 0.08	0.25 ± 0.07	0.009
DHEA	5.51 ± 2.16	4.21 ± 2.04	<0.001
*Monoacylglycerols (pmol/mL)*			
2-AG	2.63 ± 1.25	2.42 ± 0.82	0.355
1-OG	425 ± 35	423 ± 38	0.828
2-OG	75 ± 30	68 ± 18	0.189
1-LG	79 ± 19	76 ± 14	0.378
2-LG	24 ± 11	21 ± 8	0.279

Data are mean ± SD, *n* = 27. *p* value before vs. after VLCD based on a paired sample t-test

*1-LG* 1-linoleoyl glycerol, *1-OG* 1-oleoyl glycerol, *2-AG* 2-arachidonoyl glycerol, *2-LG* 2-linoleoyl glycerol, *2-OG* 2-oleoyl glycerol, *AEA* anandamide, *Alpha-LEA* *N*-α-linolenylethanolamide, *DEA* *N*-docosatetraenoylethanolamide, *DGLEA* dihomo-γ-linolenoyl ethanolamide, *DHEA* *N*-docosahexaenoylethanolamide, *EPEA* eicosapentaenoyl ethanolamide, *LEA*: *N*-linoleoylethanolamide, *OEA* *N*-oleoylethanolamide, *PDEA* *N*-pentadecanoylethanolamide, *PEA* *N*-palmitoylethanolamide, *POEA* *N*-palmitoleoylethanolamide, *SEA* *N*-stearoylethanolamide

### Adipose tissue distribution

**Fig. 3:**
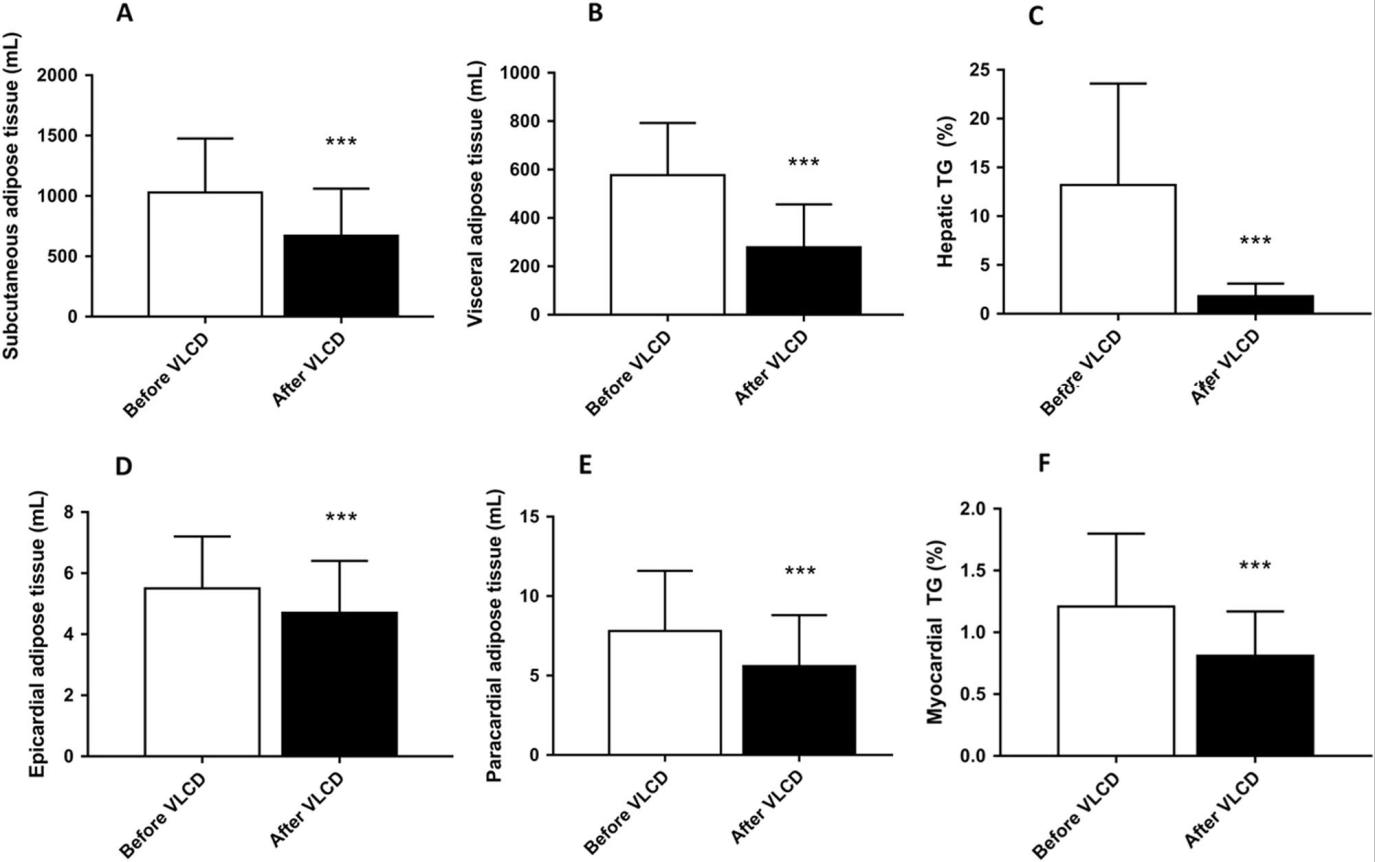
Volumes of various adipose tissue compartments before and after VLCD Change in SAT volume (**a**), VAT volume (**b**), hepatic TG content (**c**), epicardial adipose tissue volume (**d**), paracardial adipose tissue (**e**) and myocardial TG content (*n* = 22) (**f**). Results are expressed as mean ± SD. *** *p* < 0.001 *vs*. before VLCD, *n* = 27, except for F, *n* = 22

VLCD reduced VAT volume (−52%, *p* < 0.001) and SAT volume (−35%, *p* < 0.001) (Fig. 3). Furthermore, VLCD substantially decreased hepatic TG content (−86%, *p* < 0.001). VLCD also reduced epicardial fat volume (−15%, *p* < 0.001) and paracardial fat volume (−28%, *p* < 0.001). Delayed enhancement MRI revealed a septal MI in five patients, which prohibited myocardial TG measurements in these patients. Myocardial TG in the other patients decreased after VLCD (−33%, *p* < 0.001). There were no differences in myocardial TG in patients with or without MI.

### Cardiovascular parameters

The changes in cardiac dimensions and function before and after 16 weeks caloric restriction are shown in Table 3. While VLCD did not influence diastolic function, as the ratio of peak filling at early diastole and diastolic atrial contraction, the downslope after early peak filling rate, and the estimated filling pressure remained unchanged, VLCD did decrease LV mass and improved systolic cardiac function, as evidenced by an increased ejection fraction. VLCD also decreased cardiac output and increased end-diastolic volume and stroke volume. Furthermore, VLCD decreased aortic PWV from 7.9 ± 1.9 m/s at baseline to 7.2 ± 1.1 m/s (*p* = 0.016) after VLCD.

**Table 3:** Cardiac dimensions and systolic and diastolic cardiac function before and after very low calorie diet

	Before VLCD	After VLCD	*p* value
*Cardiac dimensions and function*			
LV mass (g)	114 ± 27	104 ± 27	<0.001
LV mass index (g/m^2^)	53 ± 11	53 ± 12	0.418
EDV (mL)	175 ± 38	183 ± 40	0.033
EDVI (mL/m^2^)	82 ± 15	93 ± 17	<0.001
ESV (mL)	81 ± 29	82 ± 31	0.481
ESVI (mL/m^2^)	38 ± 13	41 ± 15	<0.001
LV mass/EDV	0.66 ± 0.11	0.57 ± 0.09	<0.001
SV (mL)	94 ± 18	101 ± 17	0.007
SVI (mL/m^2^)	44 ± 7	51 ± 6	<0.001
CO (L/min)	6225 ± 982	5619 ± 1410	0.029
CI (L/min/m^2^)	2921 ± 390	2846 ± 643	0.571
EF (%)	54.8 ± 8.7	56.2 ± 7.9	0.016
E/A-peak ratio	0.95 ± 0.28	1.08 ± 0.27	0.063
E deceleration (mL/s² x 10^-^³)	−2.20 ± 0.96	−1.87 ± 0.53	0.108
E/Ea	8.9 ± 5.0	6.6 ± 4.4	0.076

Data are mean ± SD, *n* = 27. *p* value before *vs*. after VLCD based on a paired sample t-test

*A* diastolic atrial contraction, *CO* cardiac output, *CI* cardiac index, *E* early diastolic filling phase, *EDV* end-diastolic volume, *E/Ea* estimate of LV filling pressure, *EF* ejection fraction, *ESV* end-systolic volume, *I* indexed for body surface area, *LV* left ventricular, *SV* stroke volume

## Discussion

In this study, we observed that a VLCD for 16 weeks decreased plasma levels of the CBR agonist AEA and other ECs we measured of the class of *N*-acylethanolamines in obese patients with T2D and established coronary atherosclerosis. Furthermore, VLCD resulted in reductions in SAT and VAT volume, reduced ectopic lipid accumulation, and improved cardiovascular function.

We show for the first time that vigorous caloric restriction with substantial weight loss and decrease of all adipose tissue depots can reduce AEA and other *N*-acylethanolamines in patients with T2D. In contrast, VLCD did not affect plasma levels of the EC 2-AG and other monoacylglycerols. Two previous studies have investigated the effect of small reductions of body weight on EC levels, both showing no effect^25, 26^. Di Marzo et al.^6^ reported decreased plasma levels of AEA and 2-AG after a one year lifestyle modification program including healthy eating and physical activity in obese men without T2D. Furthermore, the decrease in 2-AG in that study correlated positively with a decrease in VAT volume. In our study, however, effects of VLCD on 2-AG and correlations between changes in 2-AG and VAT were not observed, due to reasons unknown to us. Furthermore, in our study, AEA levels at baseline correlated strongly with SAT volume, but not with VAT volume, which to our knowledge was not described before.

It is interesting to speculate on the mechanism behind our observation that VLCD reduces AEA and other *N*-acylethanolamines. Potentially, the VLCD can have reduced the availability of biosynthetic precursors, thereby explaining decreased levels of *N*-acylethanolamines. Furthermore, the reduced volume of adipose tissue, being a source of EC production, could explain reduced AEA levels. Also, we cannot exclude that decrease or discontinuation of use of insulin injections and glucose lowering drugs has affected plasma levels of the measured lipid species. However, in our opinion, it is likely that VLCD influenced the activity or expression of enzymes responsible for degradation of ECs. After all, it is known that in adipocytes from normoglycemic non-obese subjects the expression of fatty acid amide hydrolase (FAAH), the enzyme responsible for degradation of mainly AEA and other *N*-acylethanolamines, is increased upon stimulation with insulin^39^. Furthermore, it has been shown in vivo that insulin functions as a negative regulator of AEA levels in non-diabetic subjects, but not in insulin-resistant individuals, probably due to lack of stimulation of expression of FAAH in adipocytes^40^. It is thus conceivable that the decrease in adipose tissue volume as induced by VLCD in our study improves insulin sensitivity of adipocytes thereby increasing FAAH expression and decreasing plasma levels of AEA and other *N*-acylethanolamines. This hypothesis is supported by our unpublished data showing a higher gene expression of FAAH in SAT in normal glucose tolerant subjects than in subjects with T2D (Van Eyk, unpublished). The fact that FAAH degrades *N*-acylethanolamines but not monoacylglycerols^41^, may explain why VLCD does not reduce monoacylglycerols including 2-AG.

We also detected a decreased LV mass and heart rate after VLCD, which can be considered beneficial, since both are recognized as important predictors for cardiovascular disease^42, 43^. Furthermore, the ejection fraction increased after VLCD, which was not observed in other studies with a similar approach^27, 44^. The improvement of ejection fraction in the current study is clinically very relevant, since LV ejection fraction is an important predictor of mortality in T2D patients^45^. Moreover, we show that parameters of LV function also improve in patients with complicated T2D, corroborating previous observations in uncomplicated T2D^27^. Other investigators observed an improved LV systolic function after weight loss, though based on other parameters than an improved ejection fraction^46, 47^. However, those studies differed considerably from our study, as not all patients had T2D and/or CAD. Although the estimate of the LV filling pressure, did not change after the diet, the increased end-diastolic volume at similar estimates of LV filling pressure suggests an improved LV compliance^48^. Diastolic function did not change after VLCD in contrast to previous studies in uncomplicated T2D that found significant improvements in the ratio between peak filling at early diastole and diastolic atrial contraction after prolonged caloric restriction^27^. Our study showed a decrease in PWV, a surrogate marker for arterial stiffness and a powerful independent predictor of cardiovascular events, indicating a less stiff aorta^49^. No prior studies have been published on the effects of dietary intervention on PWV in complicated T2D.

Because ECs have various metabolic effects, including increase of food intake and lipogenesis and reduction of energy expenditure, the reduction of AEA may have contributed to the metabolic improvements and may have amplified the reduction of adipose tissue volume we observed. This way, the positive correlation between baseline plasma AEA levels and SAT volume suggests that decreased AEA levels might have contributed to reduced fat accumulation in SAT, although we were unable to detect a positive correlation between the changes of the variables, possibly related to insufficient power. Furthermore, Batetta et al.^23^ reported reductions of EC levels after dietary intervention with long-chain PUFA in Zucker rats, accompanied with reductions of ectopic lipid deposition in liver and heart. Therefore, the reduction in AEA may have contributed to the decrease in myocardial TG content, which is known to be associated with impaired myocardial function^15^. Also, it was recently shown that increased EC levels are associated with impaired coronary circulatory function and even more importantly, that gastric bypass-induced weight loss reduced EC levels and beneficially affected coronary circulatory function^24, 50^, which suggests that the decrease in AEA might also have contributed to the improved cardiac function we observed.

The associative nature of our study does not allow to establish a causal relationship between the reduction in EC levels and cardiometabolic phenotype, including weight loss, ectopic lipid deposition, or measures of cardiac function. However, considering the remarkable beneficial effects of the CB1R inverse agonist rimonabant on body weight and lipid levels^19, 20^, it is likely that the EC system is an important player in metabolism. This makes a role for the EC system in the metabolic improvements we observed plausible. Future studies specifically targeting the EC system with dietary or pharmacological interventions can provide further knowledge and help entangle the role of ECs in the development of disease. Also, studying short term effects of caloric restriction, before weight loss has occurred, on EC levels could provide more information about the mechanism. Importantly, we show that prolonged caloric restriction, regardless of the role of ECs, improves cardiac function. Direct and indirect beneficial effects of caloric restriction and weight loss may have contributed to improved cardiac function, such as decreased blood pressure, improved glycemic control and improved PWV.

A strength of this study is that it is the first to investigate the effects of prolonged caloric restriction on EC levels in relation to cardiovascular function and ectopic fat distribution in overweight T2D patients with established CAD. Furthermore in a subgroup, it was established that study parameters did not change without dietary intervention. A limitation to this study is that patients followed a diet with very low caloric content and specific composition. It thus remains to be determined whether similar effects would be obtained by other interventions that induce similar weight loss. Another limitation is that due to practical issues patients were studied after ≥5 h of fasting, which may be too short for some participants to reach baseline metabolic state. However, to reliably compare intra-individual data, we studied each patient at subsequent visits after the same fasting time. Finally, we investigated circulating ECs only, because EC concentrations, receptor expression and enzymes that play a role in synthesis and degradation of ECs within metabolic tissues could for obvious reasons not be investigated.

In conclusion, we showed that caloric restriction superposed on optimal pharmacological therapy for glucoregulation improves cardiovascular function in patients with advanced T2D. Since T2D is associated with increased cardiovascular mortality, the results of this study are highly clinically relevant. Considering the role of ECs in metabolism, we anticipate that the reduction in AEA may contribute to the improved metabolic phenotype induced by weight loss. However, mechanistic studies will have to be performed to establish the causal role of dysregulation of the EC system in ectopic lipid deposition and CAD.

## Electronic supplementary material

Supplementary tables

Text summary of content of Supplementary information
