# Supplementary material for: Caloric restriction lowers endocannabinoid tonus and improves cardiac function in type 2 diabetes
Source: Nutr Diabetes. 2018 Jan 17;8:6. doi: 10.1038/s41387-017-0016-7 (PMC5851430; doi:10.1038/s41387-017-0016-7)
Supplement: Supplementary file 1 — Supplementary tables [file 41387_2017_16_MOESM1_ESM.docx]

**Supplementary tables**

| **Suppl. Table 1.** Clinical and metabolic characteristics of a subgroup of patients four months before and at the start of the very low calorie diet. | | | |
| --- | --- | --- | --- |
|  | 4 months before VLCD | At start of VLCD | *P* value |
| ***Clinical characteristics*** |  |  |  |
| Age (years) | 63.8 ± 4.8 |  |  |
| Males, n (%) | 12 (92%) |  |  |
| T2D duration (years) | 7.8 ± 4.1 |  |  |
| Patients on insulin, n (%) | 3 (23%) | 4 (31%) |  |
| Insulin dose (units/day) | 68 ± 24 | 70 ± 22 |  |
| Body weight (kg) | 94.9 ± 10.8 | 95.5 ± 11.7 | 0.316 |
| BMI (kg/m^2^) | 31.7 ± 3.9 | 31.9 ± 4.1 | 0.328 |
| Systolic blood pressure (mmHg) | 149 ± 14 | 143 ± 12 | 0.212 |
| Diastolic blood pressure (mmHg) | 87 ± 9 | 84 ± 11 | 0.129 |
| Heart rate (beats/min) | 67 ± 15 | 63 ± 13 | 0.276 |
|  |  |  |  |
| ***Metabolic characteristics*** |  |  |  |
| Glucose (mmol/L) | 6.8 ± 1.4 | 7.4 ± 1.8 | 0.203 |
| HbA_1c_ (%) | 7.2 ± 1.5 | 6.8 ± 1.1 | 0.422 |
| HbA_1c_ (mmol/mol) | 55 ± 16 | 51 ± 12 | 0.422 |
| Triglycerides (mmol/L) | 2.01 ± 1.02 | 2.14 ± 1.00 | 0.600 |
| Total cholesterol (mmol/L) | 4.26 ± 0.76 | 4.42 ± 0.80 | 0.404 |
| HDL-cholesterol (mmol/L) | 1.15 ± 0.31 | 1.17 ± 0.30 | 0.604 |
| LDL-cholesterol (mmol/L) | 2.19 ± 0.64 | 2.27 ± 0.78 | 0.528 |
| NEFA (mmol/L) | 0.50 ± 0.20 | 0.54 ± 0.19 | 0.473 |
| Data are mean ± SD, *n*=13. *P* value 4 months before *vs.* at the start of diet based on a paired sample t-test. | | | |

| **Suppl. Table 2.** Cardiac dimensions and systolic and diastolic cardiac function of a subgroup of patients four months before and at the start of the very low calorie diet. | | | |
| --- | --- | --- | --- |
|  | 4 months before VLCD | At start of VLCD | *P* value |
|  |  |  |  |
|  |  |  |  |
| **Cardiac dimensions and function** |  |  |  |
| LV mass (g) | 118 ± 27 | 116 ± 23 | 0.470 |
| LV mass index (g/m^2^) | 57 ± 12 | 56 ± 11 | 0.452 |
| EDV (ml) | 178 ± 40 | 178 ± 40 | 0.897 |
| EDVI (ml/m^2^) | 85 ± 15 | 85 ± 17 | 0.867 |
| ESV (mL) | 86 ± 31 | 86 ± 33 | 0.919 |
| ESVI (mL/m^2^) | 41 ± 13 | 41 ± 15 | 0.994 |
| LV mass/EDV | 0.68 ± 0.17 | 0.67 ± 0.13 | 0.571 |
| SV (mL) | 92 ± 20 | 93 ± 20 | 0.695 |
| SVI (mL/m^2^) | 44 ± 8 | 44 ± 8 | 0.739 |
| CO (L/min) | 5844 ± 834 | 5762 ± 956 | 0.701 |
| CI (L/min/m^2^) | 2793 ± 281 | 2746 ± 355 | 0.645 |
| EF (%) | 52.4 ± 9.2 | 53.0 ± 10.0 | 0.443 |
| E/A-peak ratio | 0.86 ± 0.27 | 0.93 ± 0.26 | 0.304 |
| E deceleration (mL/s²x10^-^³) | -2.10 ± 1.02 | -1.92 ± 0.94 | 0.653 |
| E/Ea | 7.2 ± 4.9 | 6.8 ± 4.4 | 0.769 |
| Data are mean ± SD, *n*=13. *P* value before *vs.* after VLCD based on a paired sample t-test. A: diastolic atrial contraction, CO: cardiac output, CI: cardiac index, E: early diastolic filling phase, EDV: end-diastolic volume, E/Ea: estimate of LV filling pressure, EF: ejection fraction, ESV: end-systolic volume, I: indexed for body surface area, LV: left ventricular, SV: stroke volume. | | | |

**Suppl. Table 3.** Correlations between diet-induced changes in the endocannabinoids AEA and 2-AG and either adipose tissue volume or cardiac function.

|  | Δ plasma AEA | |  | Δ plasma 2-AG | |
| --- | --- | --- | --- | --- | --- |
|  | R^2^ | *P* value |  | R^2^ | *P* value |
|  |  |  |  |  |  |
| **Adipose tissue depots** |  |  |  |  |  |
| Δ SAT volume | 0.0625 | 0.209 |  | 0.0467 | 0.279 |
| Δ VAT volume | 0.0190 | 0.493 |  | 0.0493 | 0.265 |
| Δ Epicardial adipose tissue volume | 0.0036 | 0.768 |  | 0.0114 | 0.596 |
| Δ Paracardial adipose tissue volume | 0.0086 | 0.645 |  | 0.0035 | 0.769 |
| Δ Hepatic TG content | 0.0132 | 0.568 |  | 0.0328 | 0.365 |
| Δ Myocardial TG content | 0.0154 | 0.581 |  | 0.0829 | 0.194 |
|  |  |  |  |  |  |
| **Cardiac dimensions and function** |  |  |  |  |  |
| Δ LV mass | 0.0017 | 0.841 |  | 0.0420 | 0.306 |
| Δ EDV | 0.0365 | 0.340 |  | <0.0001 | 0.988 |
| Δ ESV | 0.1225 | 0.073 |  | 0.0058 | 0.707 |
| Δ SV | 0.0030 | 0.784 |  | 0.0029 | 0.788 |
| Δ CO | 0.0071 | 0.676 |  | 0.0199 | 0.482 |
| Δ EF | 0.0718 | 0.176 |  | <0.0001 | 0.965 |
| ΔE/Ea | 0.0017 | 0.843 |  | 0.0524 | 0.260 |
|  |  |  |  |  |  |
| Values result from correlation analyses using data from all individuals (n=27). CO: cardiac output, E/Ea: estimate of LV filling pressure, EDV: end-diastolic volume, EF: ejection fraction, ESV: end-systolic volume, LV: left ventricular, SV: stroke volume. | | | | | |
