# Supplementary material for: Caloric restriction lowers endocannabinoid tonus and improves cardiac function in type 2 diabetes
Source: Nutr Diabetes. 2018 Jan 17;8:6. doi: 10.1038/s41387-017-0016-7 (PMC5851430; doi:10.1038/s41387-017-0016-7)
Supplement: Supplementary file 2 — Text summary of content of Supplementary information [file 41387_2017_16_MOESM2_ESM.docx]

**Text summary of content of supplementary information**

Supplementary table 1 contains data on clinical and metabolic parameters of the subgroup of 13 patients that was studied 16 weeks prior to the start of the VLCD showing these variables did not change during this period.

Supplementary table 2 contains data on MR parameters of the subgroup of 13 patients that was studied 16 weeks prior to the start of the VLCD showing no significant variability before start of VLCD.

Supplementary table 3 contains data on correlations between diet-induced changes in the endocannabinoids AEA and 2-AG and either adipose tissue volume or cardiac function.
